# Detection of *suid herpesvirus 1* infectivity in pigs by propidium monoazide-qPCR

**DOI:** 10.3389/fvets.2022.975726

**Published:** 2022-10-28

**Authors:** Liu Yang, Yunzhi Long, Qianqian Li, Wenbo Song, Ying Huang, Gong Liang, Daobing Yu, Mingguang Zhou, Gaoyuan Xu, Yao Chen, Chao Huang, Xibiao Tang

**Affiliations:** ^1^Diagnostic Center Department, Wuhan Keqian Biology Co., Ltd, Wuhan, China; ^2^Veterinary Services Centre, Ezhou City Animal Husbandry Bureau, Ezhou, China

**Keywords:** propidium monoazide-qPCR, *suid herpesvirus 1*, infectious, inactivation, UV

## Abstract

At present, there is no effective experimental method for detecting whether the *suid herpesvirus 1* (*SHV-1*) detected in pigs is infectious. Although the technique of quantitative polymerase chain reaction (qPCR) has significantly improved the detection rate and accuracy of the disease, it does not differentiate between infective and non-infective status of the virus. Propidium monoazide (PMA) is a dye that can be combined with DNA molecules. The decomposition of PMA produces an azene compound covalently crosslinked with DNA molecules, thereby inhibiting PCR amplification of DNA. In this study, the combination of PMA and qPCR was used to determine the infectivity of *SHV-1*. We optimized the method from the selection of primers, the working concentration of PMA, and the method of inactivation using UV or heat inactivation. We found that when specific primer 1 was used and a PMA working concentration was 50–100 μM, heat inactivation was able to distinguish whether *SHV-1* was infectious or not. We also showed that UV prevented the virus from replicating, it did not destroy the capsid of the virus, and therefore, PMA cannot enter the virus and bind to the nucleic acid of the virus. Consequently, there is no way to identify the infectivity of the virus using UV inactivation. The study showed that the method was stable and the detection rate reached 96%. In conclusion, this method exhibited strong specificity and high sensitivity and can identify the infectivity of *SHV-1*. This method has practical significance for clinical virus isolation and the effects of disinfection of farms.

## Introduction

*Pseudorabies* (*PR*) is caused by the *suid herpesvirus 1* (*SHV-1*), a common infectious disease in animals (1). Pigs are the natural host of this virus, which invades the nervous and reproductive systems as well as leads to reproductive problems in breeding pigs (2). The virus is difficult to eliminate from farms (3) and causes high economic losses (4). Since 2011, the emergence and widespread prevalence of mutant *SHV-1* have seriously restricted the development of the pig industry (5–7). Recent epidemiological surveys of the *PR* infection rate in China demonstrated that *SHV-1* prevention and treatment require ongoing effort (8). In 16,256 tissue samples collected from 27 provinces in China, the positive rate of *SHV-1* was close to 8.0% (8). Thus, timely, rapid, and accurate early diagnosis of *SHV-1* infection is an important prerequisite for effective prevention and control of pseudorabies in pigs (9).

The main methods used to diagnose *SHV-1* include virus isolation and identification, serological testing, and molecular biology diagnosis (9). Serological testing includes neutralization, latex agglutination, immunofluorescence, and enzyme-linked immunosorbent tests (10). Molecular biology diagnosis methods include nucleic acid probe technology, polymerase chain reaction (PCR) technology, and gene chip technology (10). Quantitative PCR (qPCR) can accurately and quantitatively detect both early and latent infection and has been widely used to evaluate animal disease detection (11–13). However, qPCR does not distinguish between dead and alive viruses and cannot identify virus infectivity (14). To overcome these limitations, virus samples can be preprocessed by using propidium monoazide (PMA) (14).

Propidium monoazide is a nucleic acid dye containing photoinduced azide groups that can covalently bind to the viral nucleic acids of damaged or structurally altered viral capsids (15). In viruses that are non-infectious because of capsid protein breakage, PMA binds to the viral nucleic acid to inhibit the amplification of non-infectious viral nucleic acid during the PCR process, avoiding false-positive results (16, 17). PMA has been widely used to distinguish between live and dead bacteria, fungi, or parasites (18–20), as well as infectious and non-infectious viruses (21–23). The combination of PMA and qPCR has been applied to detect food, medical, and plant pathogenic microorganisms (24–26) and thus may be applicable for detection of *SHV-1*-infective virus.

In this study, we used PMA-qPCR to determine the infectivity of *SHV-1*. Using heat inactivation as pretreatment, gE gene primers, and a PMA working concentration of 50–100 μg can detect whether *SHV-1* is infectious. PMA-qPCR method shows a detection rate of 96%, and the results were reliable. Therefore, this method is specific and sensitive and can be used to evaluate the infectivity of *SHV-1*. This method has practical significance for clinical virus isolation and for assessing the effects of disinfection efforts on farms. In this study, we combined PMA with qPCR to establish a method that can detect *SHV-1* infectivity. The method has practical implications for clinical virus isolation and assessment of the effects of disinfection work on farms.

## Materials and methods

### PMA, cell line, virus strains, and culture methods

Propidium monoazide was purchased by Biotium (40010, Biotium, Shanghai, China). The porcine kidney cell line PK-15 cell was obtained from the American Type Culture Collection (ATCC^®^ TIB-71TM, Manassas, VA, USA) and cultured in RPMI-1640 (# SH30809.01, Hyclone^®^, Logan, UT, USA) medium containing 10% heat-inactivated fetal bovine serum (# 10099-141C, Gibco^®^, Grand Island, NY, USA) and 1% penicillin/streptomycin (# SV30010, Hyclone^®^, Logan, UT, USA) at 37°C and 5% CO_2_. The suid herpesvirus 1 (*SHV-1*) was provided by South China Agricultural University and inoculated into PK-15 cells, followed by culture at 37°C and 5% CO_2_. After 3–4 days of culture, cell cytopathic effects (CPE) were observed. The viral liquid was filtered and then diluted by 10-fold.

### *SHV-1* virus inactivation by heat or UV

The virus was boiled in a water bath for 30 min for heat inactivation. For UV inactivation, the virus suspension (1 ml) was evenly spread on the bottom of a 10-mm petri dish and exposed to ultraviolet radiation for 4 h. The UV source was a Xin Yate ZWS-type UV ultraviolet lamp (power 8 W, batch number 180818V), purchased from Suzhou Xin Yate Light Source Factory. The test irradiation intensity was 90 uW/cm^2^. Inactive *SHV-1* was used to infect PK-15 cells, followed by culture in a 37°C and 5% CO_2_ incubator for 3–4 days, after which the cells were observed for cytopathic effects. The infective virus was used to infect PK-15 cells as a positive control, and phosphate-buffered saline (PBS) was used as a negative control.

### PMA treatment

Propidium monoazide (1 mg) dye was dissolved in 500 μL of dimethyl sulfoxide (DMSO) to obtain a 4-mM storage solution, which was stored at −20°C in the dark. PMA working solution was added to the diluted virus solution to make the final concentrations of 100, 50, 20, and 10 μM and allowed to react in the dark for 13–15 min. The solution was placed under a 100-W blue light for 18–20 min and shaken once every 3–5 min.

### PMA validity detection

To determine the effectiveness of PMA, we prepared the following experimental groups based on the PMA concentration of 50 μM recommended in ref. (27). In group one, PMA working solution was added to the *SHV-1*-infective, *SHV-1*-heat-inactivated, *SHV-1*-UV-inactivated, and *SHV-1* nucleic acid solutions and PMA. In group two, PBS was added to the *SHV-1*-infective, *SHV-1*-heat-inactivated, *SHV-1*-UV-inactivated, and *SHV-1* nucleic acid solutions. Distilled H_2_O (dH_2_O) was used as a negative control in qPCR amplification. The experimental design is given in Table 1.

**Table 1:** Propidium monoazide (PMA) treatment groups and controls.

**Group one**		**Group two**		**Control**
Infective *SHV-1*	+PMA	Infective *SHV-1*	+PBS	dH_2_O
*SHV-1*-heat-inactivated		*SHV-1*-heat-inactivated		
*SHV-1*-UV-inactivated		*SHV-1*-UV-inactivated		
*SHV-1*-nucleic		*SHV-1*-nucleic		

### PMA working concentration and identification of dominant primers

To determine the PMA working concentration, the infective virus and heat-inactivated virus were treated with 100, 50, 20, and 10 μM PMA as the experimental groups. dH_2_O was included as a negative control in qPCR amplification.

Two pairs of primers were designed according to the envelope glycoprotein E (gE) gene of *SHV-1* reported in the NCBI, and different primers were screened. The primer sequences and amplicon lengths are listed in Table 2.

**Table 2:** Primers.

**Primer name**	**5^′^−3^′^sequence**	**Product length (bp)**
*SHV-1*-gE- F1	TTTGGATCCATGCGGCCCTTTCTG	384
*SHV-1*-gE- R1	TTTGAATTCTTACGACACGGCGTCGCA	
*SHV-1*-gE-F2	TTTGGATCCATGCGGCCCTTTCTG	276
*SHV-1*-gE-R2	TTTGAATTCTTACGACACGGCGTCGCA	

To verify the specificity of the primer amplification products, the two pairs of specific primers designed were identified by qPCR for the SHV-1 virus, with dH_2_O used as a negative control. The reaction system contained 2 × AceQ qPCR SYBR Green Master Mix (10.0 μL), 10 μM upstream primer (0.4 μL), 10 μM downstream primer (0.4 μL), 50 × ROX Reference Dye 1 (0.4 μL), and template cDNA (2.0 μL), and deionized water was added to a total reaction volume of 20 μL. The reaction conditions were as follows: pre-denaturation at 95°C for 5 min, 40 cycles at 95°C for 10 s and 60°C for 30 s, followed by 95°C for 15 s, 60°C for 60 s, and 95°C for 15 s. A CFX96TM real-time PCR instrument (Bio-Rad, Hercules, CA, USA) was used for this procedure.

After the virus solution was treated with different concentrations of PMA, the Ct value of the virus samples was quantified by qPCR using two pairs of gene-specific primers.

### Characteristics and application of *SHV-1* PMA-qPCR

The *SHV-1*-gE gene fragment amplified by primer 1 was inserted into pUC19 to form a recombinant plasmid. Recombinant pUC19-primer 1 was extracted using a Qiagen Plasmid Plus Kit (Qiagen, Hilden, Germany) and stored at −20°C. The plasmid concentration was measured, and a 10-fold dilution of this sample was used for qPCR.

To test the specificity of the primer, the nucleic acids of *SHV-1*, swine fever virus, porcine reproductive and respiratory syndrome virus, porcine circovirus, and porcine epidemic diarrhea virus were extracted and verified by qPCR. dH_2_O was included in the qPCR step as a negative control.

To clarify the effective concentration range for successfully detecting *SHV-1*, the virus was diluted by 10-, 100-, and 1,000-fold and amplified with the appropriate primer and PMA concentrations using qPCR. dH_2_O was included in the qPCR step as a negative control.

### Validation of PMA-qPCR method

To verify whether PMA-qPCR can detect *SHV-1* infectivity, we used this method to evaluate 50 infective *SHV-1* virus samples and 50 inactivated *SHV-1* copies. PBS was set as the negative control group. dH_2_O was included in the qPCR step as a negative control.

### Data processing

A two-way analysis of variance was used to assess differences between groups, and Pearson's correlation coefficient (GraphPad Prism 5.0, GraphPad, Inc., La Jolla, CA, USA) was used for correlation analysis when required. The results were judged as follows: For a sample Ct value ≤ 35, the sample was judged as positive; 35 < sample Ct value ≤ 40, if the amplification curve was logarithmic, the sample was judged as suspiciously positive and otherwise the sample was judged as negative; when the sample was suspected as positive, it was retested. If the Ct value of the retested sample was ≤ 35, the sample was judged as positive; otherwise, the sample was judged as negative. When the sample had no Ct value or if the Ct > 40, the sample was judged as negative.

## Results

### *SHV-1* was inactivated by heat and UV

**Figure 1:**
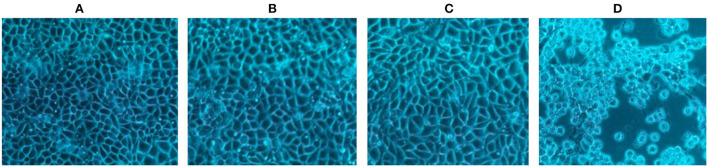
The CPE results of different inactivation methods. **(A)** CPE diagram of heat-inactivated cells; **(B)** CPE diagram of UV-inactivated cells; **(C)** blank group cell diagram; **(D)** CPE diagram of non-inactivated cells.

Group one contained cells infected with, group two contained cells infected with, group three contained cells uninfected with *SHV-1* (blank control group), and group four contained cells infected with *SHV-1* (cytopathic group). The heat-inactivated and UV-inactivated groups and blank control exhibited no cytopathic effects (Figures 1A–C), whereas the non-inactivated group exhibited clear cytopathic effects (Figure 1D). Thus, *SHV-1* can be inactivated by heat and UV treatments.

### Effectiveness of PMA for detecting *SHV-1* infection verified using qPCR

**Figure 2:**
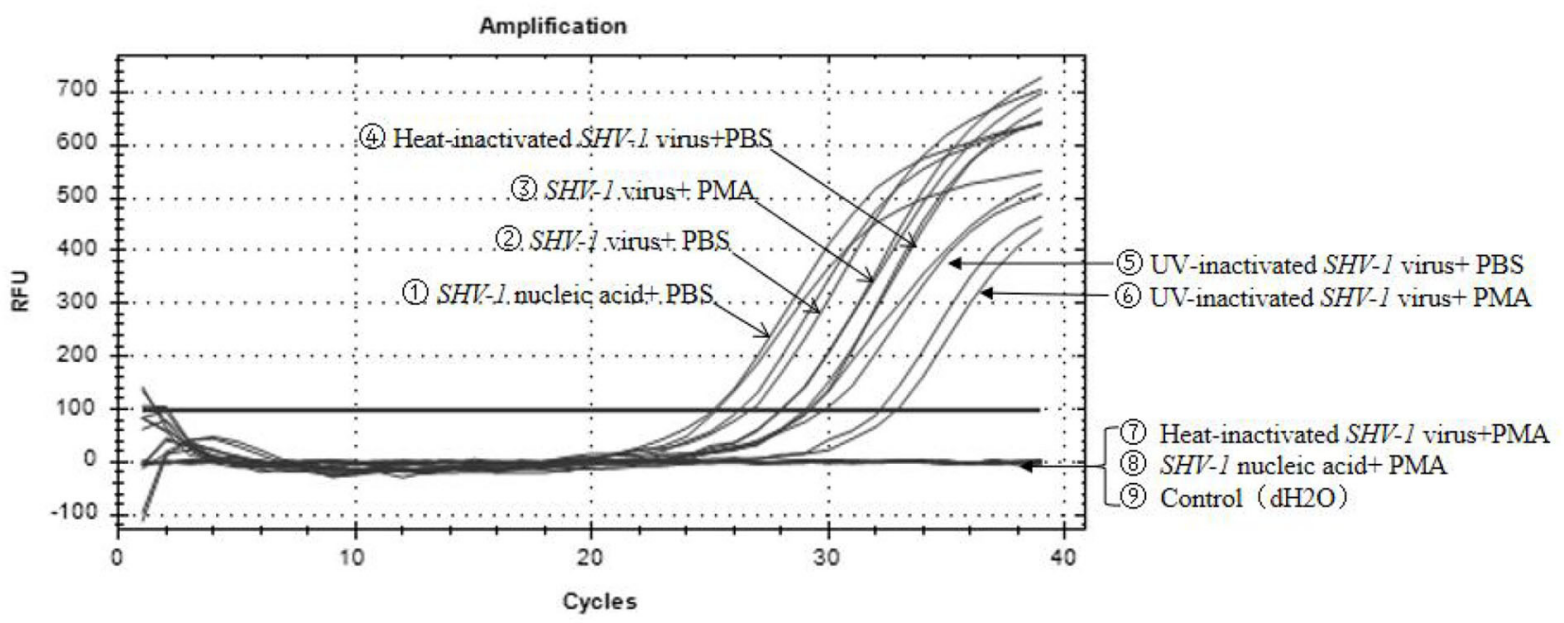
Effect of PMA on qPCR detection of *SHV-1*. ① Infective *SHV-1* virus + PMA group; ② infective *SHV-1* virus + PBS group; ③ heat-inactivated *SHV-1* virus + PBS group; ④ UV inactivation *SHV-1* virus + PMA group; ⑤ UV inactivation *SHV-1* virus + PBS group; ⑥ *SHV-1* virus nucleic acid + PMA group; ⑦ *SHV-1* virus nucleic acid + PBS group; ⑧ heat-inactivated *SHV-1* virus + PMA group; ⑨ control group (dH_2_O).

Propidium monoazide was added to *SHV-1* after heat and UV treatments, whereas PBS was added to the control group. The results are shown in Figure 2. Heat-inactivated virus + PMA group, *SHV-1* nucleic acid + PMA group, and the negative control group had no copy number. The Ct values of the other treatment groups were all ≤ 35 and the copy numbers range from 1,400 to 95,000/μL, indicating positive results. PMA has no effect on the qPCR detection of infective *SHV-1* virus. Moreover, PMA can bind to nucleic acid of *SHV-1* affecting qPCR amplification. UV inactivation has no effect on PMA detection. The specific results are given in Table 3.

**Table 3:** PMA validity detection.

**Group**	**CT**
*SHV-1* nucleic acid + PBS	25.1 ± 0.08
*SHV-1* virus + PBS	26.4 ± 0.33
*SHV-1* virus + PMA	27.9 ± 0.07
Heat-inactivated *SHV-1* virus + PBS	29.0 ± 0.12
UV-inactivated *SHV-1* virus + PBS	29.4 ± 0.39
UV-inactivated *SHV-1* virus + PMA	32.5 ± 0.47
Heat-inactivated *SHV-1* virus +PMA	N/A
SHV-1 nucleic acid + PMA	N/A
Control (dH_2_O)	N/A

### PMA working concentration and primer evaluation

**Figure 3:**
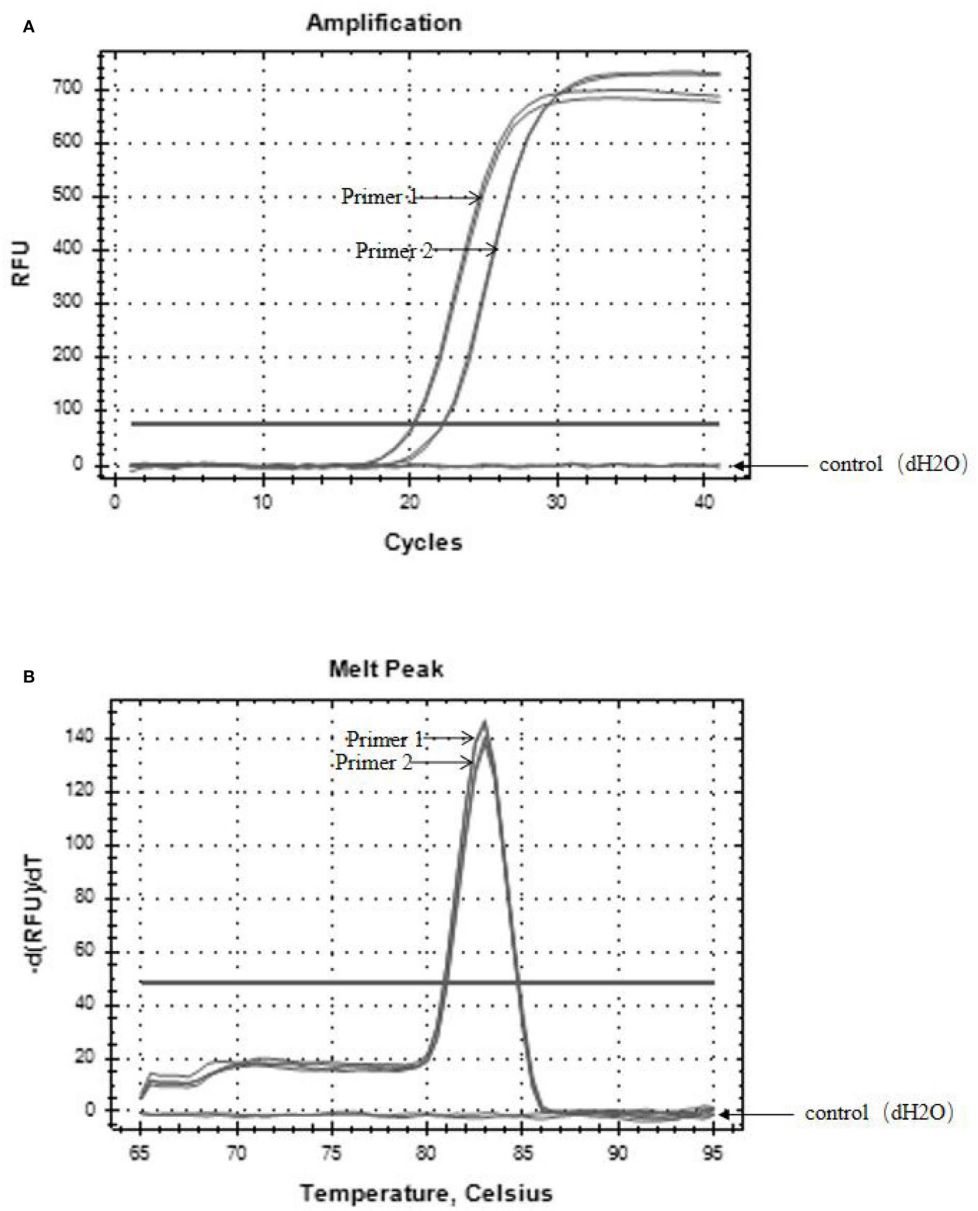
The results of primer amplified products. **(A)** Amplification curves of two primer pairs; **(B)** melting curves of two primer pairs. The control group was dH_2_O.

The specific results of the amplification products of the two pairs of primers are shown in Figure 3, which are the amplification curve (Figure 3A) and the melting curve (Figure 3B). The CT values of the two groups of primers were 20 and 22, respectively, and the corresponding copy numbers were 1.7^*^10^7^ copies/μL and 5.5^*^10^5^ copies/μL, which were positive results. Only a single peak was generated at 80–85°C. It indicated that both primers could specifically amplify the target band of *SHV-1*.

**Figure 4:**
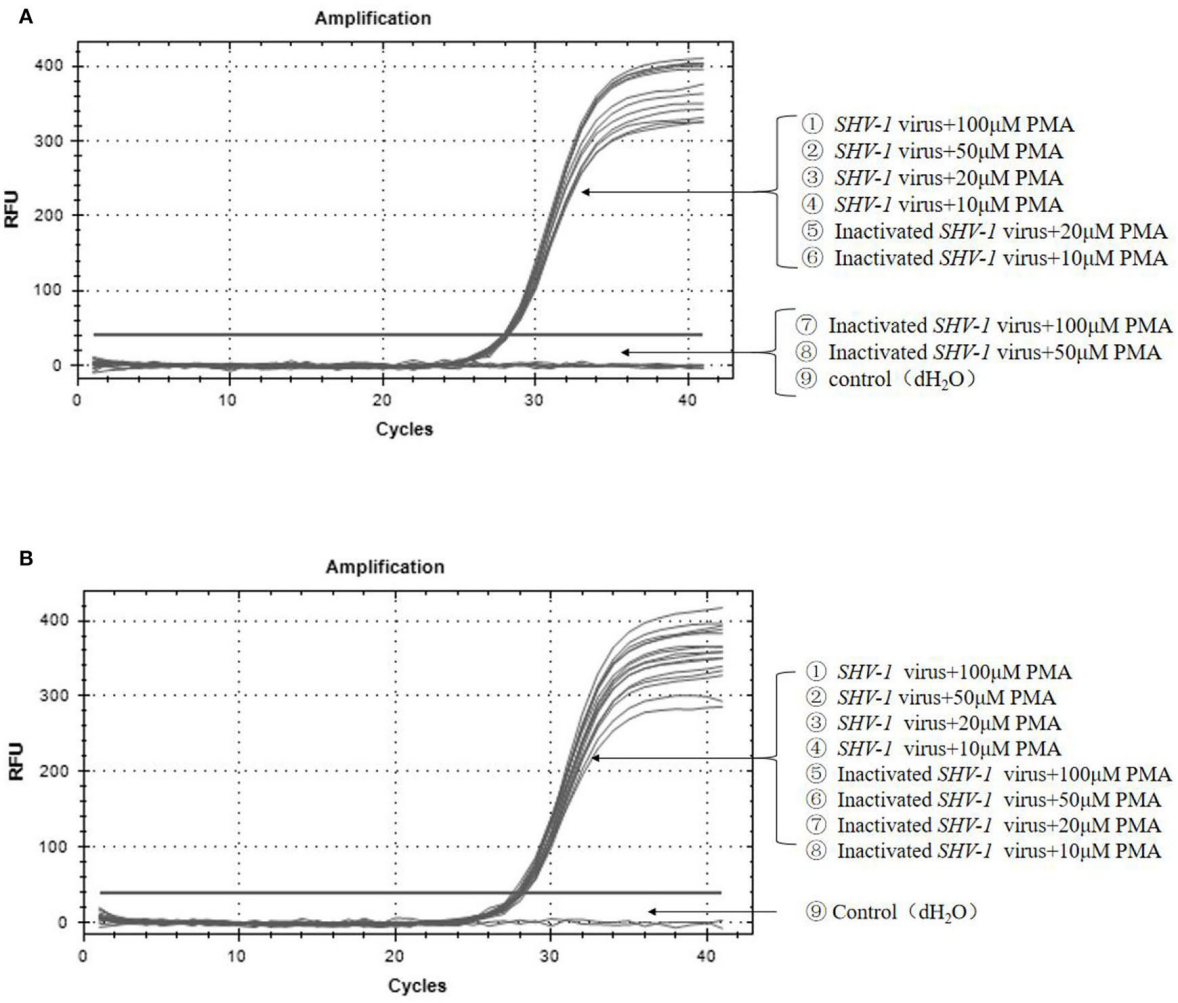
Effectiveness of different virus dilutions on *SHV-1* PMA-qPCR. **(A)** Different PMA concentrations on *SHV-1* virus qPCR. ①-④ the different concentration PMA + *SHV-1* virus group; ⑤-⑧ the different concentration PMA + inactivated *SHV-1* virus group; ⑨ the control group (dH_2_O); **(B)** different primer pairs PMA-qPCR of porcine *SHV-1* virus. ①-④ the different concentration PMA + *SHV-1* virus group; ⑤-⑧ the different concentration PMA + inactivated *SHV-1* virus group; ⑨ the control group (dH_2_O).

To further confirm the primers, infective and heat-inactivated viruses were treated with PMA concentrations of 100, 50, 20, and 10 μM, and primers 1 (Figure 4A) and 2 (Figure 4B) were used for qPCR to establish the dominant primers and effective PMA concentrations. For primer 1, when the final concentration of PMA was 10 or 20 μM, the Ct values of infective and inactivated *SHV-1* viruses amplified by PMA-qPCR were about 28, the corresponding copy numbers were about 1.8^*^10^4^ copies/μL, and thus infective and inactivated *SHV-1* virus could not be distinguished. When the final concentration of PMA was 50 or 100 μM, the Ct values of *SHV-1* amplified by PMA-qPCR were about 28 and the corresponding copy numbers were about 1.8^*^10^4^ copies/μL, indicating positive detection. Inactivated *SHV-1* did not exhibit a Ct value and had no copy number. At final PMA concentrations of 50 and 100 μM, the primers were used for qPCR to distinguish infective from inactivated *SHV-1*. For primer 2, except for the control group, the Ct values of each group were about 28 and the corresponding copy numbers were about 1.8^*^10^4^ copies/μL, indicating that the final PMA concentration did not affect the detection of infective and inactivated *SHV-1* and could not determine whether *SHV-1* was infectious. The specific results are given in Table 4.

**Table 4:** Two primers to determine the working PMA concentrations.

**PMA (μM)**	**Primer 1**		**Primer 2**	
	***SHV-1* virus**	***SHV-1* inactivated virus**	***SHV-1* virus**	***SHV-1* inactivated virus**
100	28.5 ± 0.08	N/A	28.4 ± 0.09	28.3 ± 0.02
50	28.9 ± 0.01	N/A	28.5 ± 0.28	28.7 ± 0.08
20	28.5 ± 0.30	28.8 ± 0.08	28.2 ± 0.15	28.3 ± 0.32
10	28.3 ± 0.28	28.3 ± 0.08	28.7 ± 0.02	28.4 ± 0.06

### Primer 1 showed high specificity and sensitivity for detection of *SHV-1*

**Figure 5:**
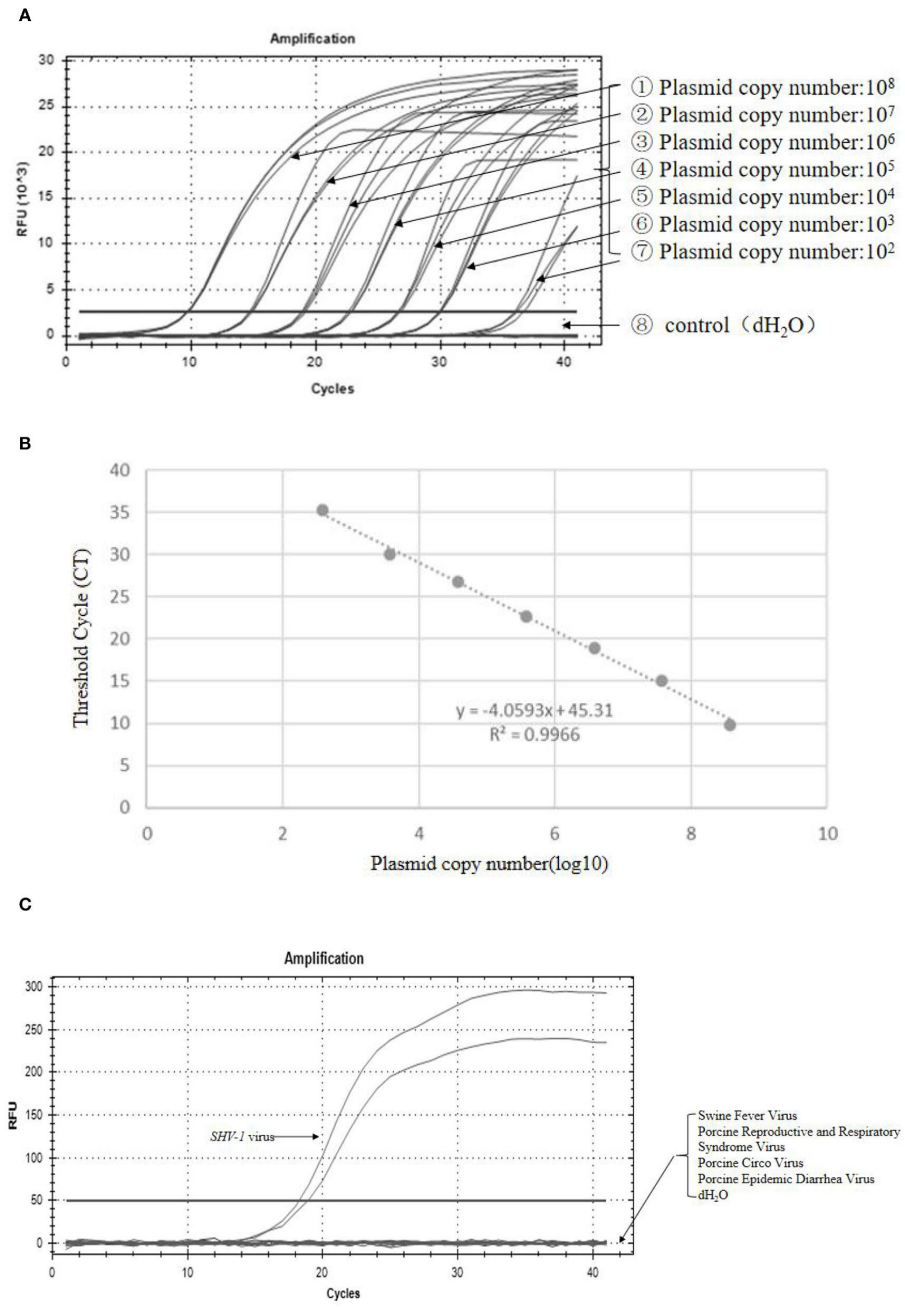
Primer 1 showed high specificity and sensitivity for detection of *SHV-1*. **(A)** The amplification curve was established with the recombinant plasmid pUC19-SHV-1-gE standard; **(B)** standard curve line, where y represents the Ct value and *x* represents the logarithm of the gene copy number concentration; *R*^2^ = 0.9966; **(C)** fluorescence quantification of different viruses. The control group was swine fever virus, porcine reproductive and respiratory syndrome virus, porcine circovirus, porcine epidemic diarrhea virus, and dH_2_O.

The recombinant plasmid PUC19-*SHV-1*-GE was constructed to determine the sensitivity of primer 1. The amplification curve and regression equation (*Y* = −4.0593 *X* + 45.31, *R*^2^ = 0.9966) are presented in Figures 5A,B. To determine the specificity of the primers for *SHV-1*, different viruses were detected with primer 1 using qPCR. The *SHV-1*-infective virus group showed positive results (Figure 5C), whereas swine fever virus, porcine reproductive and respiratory syndrome virus, pig round circovirus, porcine epidemic diarrhea virus, and water showed negative results (Figure 5C). Thus, primer 1 exhibited clear specificity and sensitivity for *SHV-1*.

### Effectiveness of different virus dilutions on *SHV-1* PMA-qPCR

*Suid herpesvirus 1* was diluted 10-, 100-, and 1,000-fold and then mixed with PMA for qRCR detection. The virus stock solution, diluted virus solution, and heat-inactivated virus stock solution exhibited positive results (Table 5). The Ct values of the original, 10-fold diluted, 100-fold diluted, and 1,000-fold diluted virus solutions were 26.4, 28.0, 30.2, and 32.6, respectively. Substituting these values into the standard curve yielded copy numbers of 18,365.38, 14,454, 5,276.14, and 1,352.33 copies/μL, respectively. According to the standard curve, the initial concentration of the virus can be calculated to be about 0.03 ng/uL. This method can be used to determine the infectivity of *SHV-1* with a virus concentration of not <0.03 ng/uL and a dilution of not more than 1,000-fold.

**Table 5:** Effectiveness of different virus dilutions on *SHV-1* PMA-qPCR.

**Group**	**Ct**
*SHV-1* virus stock	26.4 ± 0.13
*SHV-1* virus 10-fold dilution	28.0 ± 0.49
*SHV-1* virus 100-fold dilution	30.2 ± 0.19
*SHV-1* virus 1000-fold dilution	32.6 ± 0.12
*SHV-1* inactivated virus stock	33.4 ± 0.23
*SHV-1* inactivated virus 10-fold dilution	N/A
*SHV-1* inactivated virus 100-fold dilution	N/A
*SHV-1* inactivated virus 1000-fold dilution	N/A
Control (dH_2_O)	N/A

### Infectivity of *SHV-1* detected using PMA-qPCR

**Figure 6:**
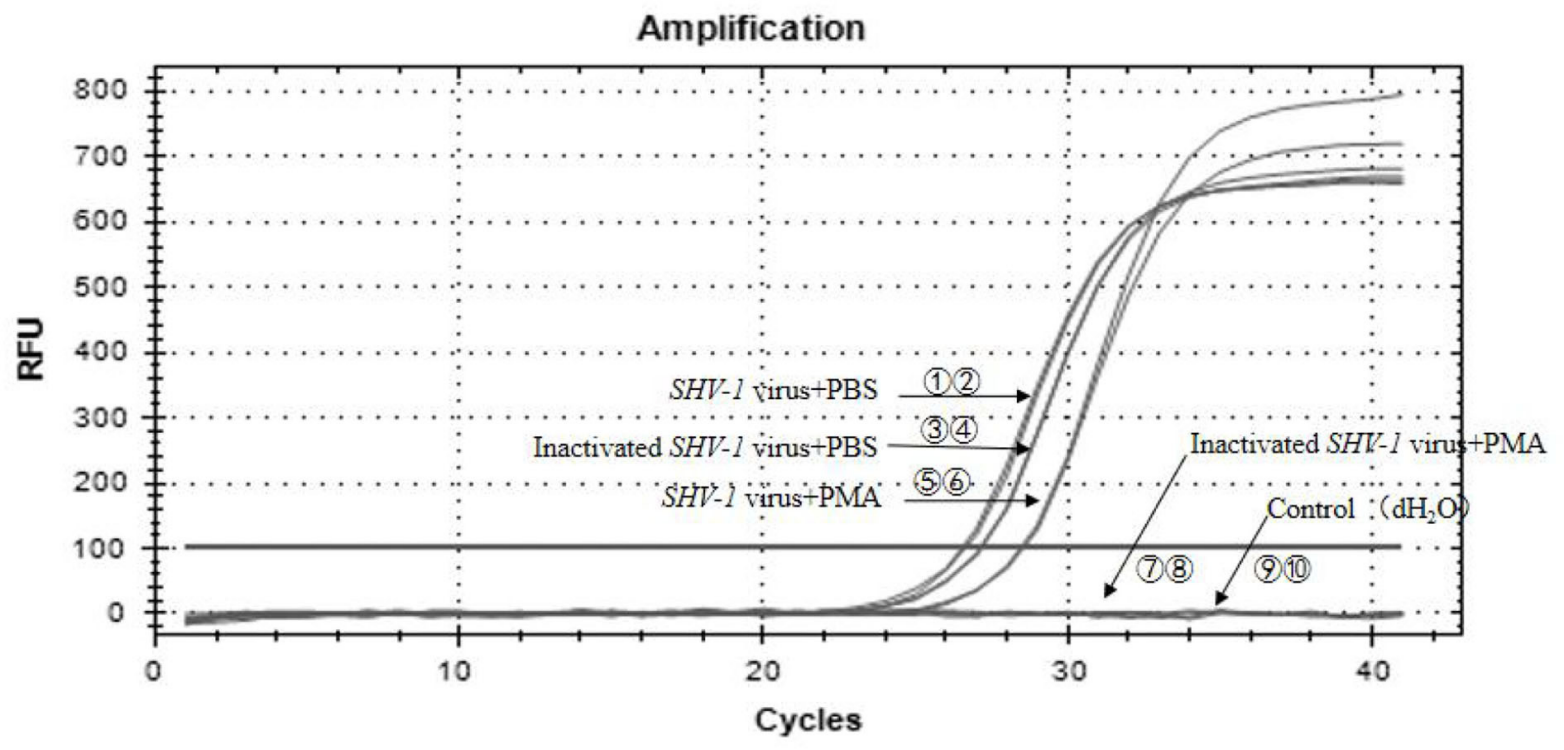
PMA-qPCR of infectious SHV-1 virus. ①,② The *SHV-1* virus + PBS group (sample no. 47); ③,④ the inactivated virus + PBS group (sample no. 65); ⑤,⑥ the *SHV-1* virus + PMA group (sample no. 6); ⑦,⑧ the inactivated virus + PMA group (sample no. 51); ⑨,⑩ the control group (dH_2_O).

In the PMA treatment group, of the 50 inactivated virus samples, 48 samples showed negative Ct values >35, indicating negative results. The Ct values of the 50 infective samples ranged from 26 to 30, corresponding to copy numbers of 6^*^10^3^-6^*^10^4^ copies/uL, indicating positive results. The Ct values of the inactivated and infective virus samples in the PBS group were all <35, indicating positive results, although we could not distinguish whether the virus was infective or not. The PMA-qPCR method showed a detection rate of 96%, and the results were reliable. The results for all samples are given in Table 6. *SHV-1* was detected using PMA-qPCR, and the partial amplification is shown in Figure 6. The Ct values of serial numbers ①-⑥ were <35, indicating that all samples were positive, including the infective virus group, inactivated virus group, and infective virus + PMA group. The Ct values of serial numbers ⑦-⑩ were >35, indicating negative results, including in the inactivated virus + PMA group and control group (dH_2_O).

**Table 6:** Infectivity of *SHV-1* detected using PMA-qPCR.

**Sample/group**	**Infective *SHV-1* + PMA**	**Infective *SHV-1* + PBS**	**Sample/group**	**Inactivated *SHV-1* + PMA**	**Inactivated *SHV-1* + PBS**
1	28.4	26.7	51	–	27.5
2	28.7	26.9	52	–	28.9
3	26.8	24.1	53	–	27.8
4	27.3	23.8	54	–	26.4
5	28.1	28.9	55	–	27.3
6	28.2	29.5	56	–	28.1
7	25.3	26.0	57	–	28.2
8	25.9	26.1	58	–	29.0
9	28.7	24.5	59	–	28.4
10	29.4	25.3	60	–	28.8
11	26.1	26.5	61	–	29.7
12	27.5	26.3	62	–	28.0
13	25.7	26.3	63	–	29.3
14	26.3	26.2	64	–	28.6
15	26.7	25.3	65	–	27.2
16	26.4	26.7	66	–	27.7
17	28.3	27.5	67	–	24.3
18	26.6	28.8	68	–	28.8
19	26.5	26.6	69	–	26.0
20	27.4	26.5	70	–	27.5
21	27.1	28.7	71	–	28.8
22	28.3	29.3	72	–	29.3
23	29.1	28.8	73	–	29.6
24	26.2	26.9	74	–	28.6
25	26.5	28.2	75	–	28.3
26	26.2	25.7	76	–	29.9
27	27.7	28.5	77	–	28.8
28	26.0	26.3	78	–	28.8
29	27.4	26.1	79	–	28.9
30	28.8	27.5	80	–	29.2
31	27.3	26.1	81	–	28.6
32	27.2	25.4	82	–	29.0
33	28.6	25.1	83	–	25.6
34	25.9	25.2	84	–	29.7
35	25.5	23.9	85	–	29.8
36	29.3	24.1	86	–	29.9
37	27.8	24.1	87	–	38.5
38	27.6	28.2	88	–	28.3
39	28.3	27.8	89	–	24.5
40	26.7	28.6	90	–	25.9
41	27.3	28.1	91	–	26.4
42	26.6	29.9	92	–	28.4
43	25.4	29.0	93	–	29.7
44	28.5	27.5	94	–	26.4
45	29.4	27.9	95	–	27.5
46	29.3	24.6	96	–	27.6
47	28.8	26.7	97	–	26.8
48	29.7	28.1	98	–	29.3
49	28.4	27.6	99	32.9	25.8
50	27.9	26.9	100	34.6	27.2

## Discussion

*Pseudorabies* has caused significant economic losses to the global pig industry. *SHV-1* is prevalent in China, which severely restricts the development of the breeding industry. The timely, rapid, and accurate diagnosis of *SHV-1* infection is necessary for effective prevention and control of swine *PR*.

In the OIE Manual of OIE Handbook of Diagnostic Tests and Vaccines for Terrestrial Animals, methods for identifying *PR* mainly include pathogenic identification and serological testing. Pathogen identification shows high sensitivity and specificity and is rapid, simple, and suitable for clinical detection of *SHV-1*; however, this method cannot distinguish between live and dead viruses, that is, whether the virus is infectious. Serological testing has a high coincidence rate and specificity, making it suitable for laboratory sample inspection, quarantine and origin analysis, epidemiological investigation, and screening and establishment of healthy pigs without disease. However, this method is time- and labor-intensive. To improve detection methods, we combined PMA and qPCR, which simplified the detection process and enabled the determination of virus infectivity.

Propidium monoazide is a photosensitive dye with a high affinity for nucleic acids. When exposed to strong visible light, PMA binds covalently to DNA or RNA (28, 29). PMA has been widely used to distinguish between live and dead bacteria, fungi, or parasites (18–20), as well as infectious and non-infectious viruses (21–23). However, PMA-qPCR detection has not been used to detect *SHV-1*. Thus, we established a method for identifying *SHV-1* infectivity.

To verify the effectiveness of PMA in distinguishing *SHV-1* infectivity, we first tested the effect of PMA on *SHV-1* detection using qPCR. The results showed that the addition of PMA did not affect the *SHV-1* detection results. PMA can only penetrate viruses with damaged capsids (21) and binds irreversibly to DNA *via* photoactivation, preventing DNA amplification by PCR (19, 30). Infective viruses have intact capsid proteins, and PMA cannot react with nucleic acids through the capsid proteins; thus, live viruses with intact capsids can be distinguished from non-infectious viruses with defective capsids (28, 29). To verify this, Karim et al. (31) performed heat inactivation, UV inactivation, and chlorine inactivation of the virus and then examined virus infectivity using PMA. The results showed that PMA rt-PCR differentiated between infectious poliovirus and heat-inactivated poliovirus following heat inactivation of the virus, but not MNV-1 virus; when MNV-1 virus was inactivated at a higher CT (2.7 mg-ml/min) chlorine level, PMA rt-PCR was able to distinguish or partially distinguish infectious virus from chlorine-inactivated virus, but it is ineffective for poliovirus; in addition, PMA treatment has no obvious effect on distinguishing infectious virus from UV-inactivated virus. Chlorine inactivation primarily damages viral nucleic acids (32), but high concentrations of chlorine damage proteins (33). MNV-1 chloride inactivation results suggest that higher chloride CT is required for MNV-1 capsid damage and subsequent PMA entry and binding (33). Considering that a high chloride concentration may affect the binding of PMA (33, 34), non-infectious viruses were prepared by heat inactivation and UV inactivation in this study. The results showed that when the virus was heat-inactivated, PMA-qPCR could distinguish between infectious *SHV-1* and heat-inactivated *SHV-1*, but could not differentiate between infectious *SHV-1* and UV-inactivated *SHV-1*. The results are similar to those of the studies described above. Although UV rays prevent virus replication, PMA still cannot enter the viral capsid and bind to the viral nucleic acid (32). We showed that PMA could bind to the viral nucleic acids, making them undetectable by qPCR and thus reducing false-positive results.

It is worth noting that the CT values of different treatment groups were significantly different (Table 3), although this did not affect the judgment of the results (15). For infectious virus, the addition of PMA slightly decreased the CT value compared with the PBS group. One argument is that PMA is toxic to the virus, although the effect of this toxicity is small (21, 30). Another way of saying that the proportion of dead and alive, the more dead cells, the PMA modification of the DNA from dead cells inhibited the amplification (19), resulting in a decrease in CT value, which may be that the virus has already died before inactivation (20). For heat-killed viruses, the addition of PMA can well identify the infectivity of the disease. For UV-inactivated viruses, the addition of PMA has no effect on identifying virus infectivity and at the same time reduces the CT value to a greater extent. In addition to the effect of PMA, it is mainly due to UV damage to the nucleic acid of the virus, which inhibits qPCR amplification to a greater extent (35).

To improve the accuracy of PMA-qPCR, we designed primers for gE gene of *SHV-1* genome and screened different primers. The screening results showed that even for primers designed for the same gene, under the same PMA treatment conditions and using the same virus concentration, not all primers were equally effective. In this assay, primer 1 was able to identify infectious *SHV-1* virus and heat-inactivated *SHV-1* virus at PMA concentrations of 50–100 μM. However, primer 2 could not distinguish the infectivity of the virus at any PMA concentration. This result may be related to the size of the amplicon corresponding to the primers (36). The size of the amplicon corresponding to primer 1 and primer 2 in this experiment was 384 and 276 bp, respectively. It has been shown that an amplicon length of at least 190 bp is required for efficient pma-mediated inhibition of DNA amplification (37). When the amplicon is larger, the longer the polymerase needs to cover, the more likely the target DNA will be blocked by covalent bonds. However, too long amplicon length will also reduce the sensitivity of qPCR, and a balance needs to be found between the two.

Similarly, pretreatment with PMA was key to establishing the PMA-qPCR detection method (38). The PMA working solution and incubation time are important parameters when pretreating the virus (21). For example, when the PMA method was used to detect dead *Mycobacterium tuberculosis* bacteria, the applicable concentrations were different (30, 39, 40). This may be related to the source of the sample. In the PMA-qPCR detection method established by Wang et al., the optimal concentration of PMA was 15 ng/μL (40). In this study, we chose the optimal PMA treatment concentration of 50–100 μM.

In addition, we examined the effect of virus dilution on PMA-qPCR; the results showed that when the virus was diluted by 10–1,000-fold, it was possible to determine whether *SHV-1* was infectious with a detectable virus copy number range of 1,352.33–18,365.38 copies/μL. Thus, this method has certain requirements for the detection range of the virus. We also showed that PMA-qPCR detection can be performed to determine *SHV-1* infectivity. However, our sample size was small, and additional clinical samples will be evaluated to confirm our results. In the sample detection, it can be found that the PMA-treated group has an average increase of 0.6 CT relative to the PBS group (data not shown). According to the average CT, the loss of about 10^4^ copies due to PMA treatment can be quantified. The higher the virus concentration, the loss of more. When the virus stock solution is diluted more than 1,000 times, that is, when the virus concentration is low, the PMA concentration is relatively supersaturated, resulting in false negative results (39). It may not have been detected due to the presence of DNA or inactivated virus in the sample (20). Follow-up studies are needed to further optimize and expand the scope of virus detection and improve detection efficiency.

In summary, we used the *SHV-1* gE gene as a detection target and established a PMA-qPCR method for rapidly and quantitatively determining the infectious ability of *PR*. This method shows high specificity, sensitivity, and repeatability and can efficiently, quickly, and accurately identify *SHV-1* infection. Our PMA-qPCR method can be applied in clinical virus isolation and to establish the effectiveness of the disinfection of farms. It is critical to note that PMA treatment of samples shows promise as a method of capsid protein-damaged viral DNA amplification. However, for UV-inactivated cells, PMA treatment is ineffective (35), which poses a problem for the application of PMA. Likewise, PMA treatment had no effect on inactive cells with intact cell membranes, which were killed by mechanisms that did not directly target the cell membrane. The same goes for viruses. Therefore, other additional improvements in the application and investigation of PMA therapy are needed. Nocker and Camper (41) speculated that the use of “active labile compounds” might be a viable approach. Further research is required.

## Conclusion

A combination of PMA and qPCR was used to detect the infectivity of *SHV-1*. After confirming that the addition of PMA did not affect the detection of live viruses, we established that primer gE, a PMA concentration of 50–100 μM, and a virus solution diluted by 10–1,000-fold were optimal experimental conditions. We determined whether heat-inactivated *SHV-1* was infectious. Our method showed high specificity, sensitivity, and repeatability and can efficiently, quickly, and accurately identify *SHV-1* infectivity. Our PMA-qPCR method can be applied for clinical virus isolation and to establish the efficacy of disinfection of farms.

## Data availability statement

The original contributions presented in the study are included in the article/supplementary material, further inquiries can be directed to the corresponding authors.

## Author contributions

LY designed and conducted experiments, analyzed data, and wrote the manuscript. YL, QL, WS, YH, GL, DY, MZ, GX, and YC conducted the experiment. CH and XT participated in the experiment and conducted data analysis. All authors contributed to the article and approved the submitted version.

## Funding

This study received funding from Wuhan Keqian Biology Co., Ltd. The funder was not involved in the study design, collection, analysis, interpretation of data, the writing of this article, or the decision to submit it for publication.

## Conflict of interest

Authors LY, YL, QL, WS, YH, GL, DY, MZ, GX, CH, and XT were employed by Wuhan Keqian Biology Co., Ltd. The remaining author declares that the research was conducted in the absence of any commercial or financial relationships that could be construed as a potential conflict of interest.

## Publisher's note

All claims expressed in this article are solely those of the authors and do not necessarily represent those of their affiliated organizations, or those of the publisher, the editors and the reviewers. Any product that may be evaluated in this article, or claim that may be made by its manufacturer, is not guaranteed or endorsed by the publisher.
